# Buffalo Lithia Springs

**Published:** 1889-12

**Authors:** 


We had the pleasure of a call from the accommodating agent, Thomas W. Hood, in the interest of the "Maltine" Co. The samples handed us were beautiful, and of superior quality. This house and their goods stand deservedly high with the profession everywhere. Among them Maltine with Pepsin and Pancreatin; Maltine with Cascara; Maltine with Iron, Quinine and Strychnia, etc.

